# Evaluation of Axilla With Sentinel Lymph Node Biopsy (Using Methylene-Blue) and Reverse Axillary Mapping (Using Fluorescein) to Validate Optimum and Safe Axillary Dissection in Breast Cancer

**DOI:** 10.7759/cureus.45267

**Published:** 2023-09-14

**Authors:** Aswin Puthangot, Chintamani Chintamani, Megha Tandon

**Affiliations:** 1 Department of General Surgery, Vardhman Mahavir Medical College & Safdarjung Hospital, New Delhi, IND

**Keywords:** alnd, axillary lymph node dissection, sodium fluorescein, methylene blue dye, axillary reverse mapping, sentinel lymphnode biopsy, breast cancer

## Abstract

Introduction

Sentinel lymph node biopsy (SLNB) has replaced routine axillary lymph node dissection (ALND) in node-negative axillae. In cases where the axilla needs to be dissected, one must dissect below the uppermost intercostobrachial nerve (ICBN) to avoid damaging arm lymphatics.

Methods

One milliliter of methylene blue dye was injected around the areola. Fluorescein dye (1 ml) was injected into the upper arm. After SLNB and ALND, the axilla was visualized under blue light. The location of fluorescent lymphatics was mapped with respect to the uppermost ICBN.

Results

The identification rate of sentinel lymph nodes and arm lymphatics was 100%. Arm lymphatics were above ICBN in 86.7%. The false negative rate of SLNB was 13%, with sensitivity and specificity of 87% and 100%, respectively.

Conclusions

SLNB using the single-dye technique has results comparable to dual agent studies that utilize blue dye and radioactive colloid. The uppermost ICBN could define the superior limit of axillary dissection.

## Introduction

Breast cancer is the most common malignancy among women worldwide. The Indian Council for Medical Research (ICMR) reports 150,000 new breast cancer cases in India per year, of which 70,000 succumb every year [1].

There has been a paradigm shift in the management of breast cancer over the last few decades. Although surgery has remained the cornerstone of management, the approach has become more conservative. In breast surgery, we have moved from radical surgeries like mastectomy to breast conservation [2]. For the axilla, axillary lymph node dissection (ALND) has been the standard of care. However, this procedure is now reserved only for node-positive axillae due to associated morbidities of lymphedema, neuropathy, and shoulder rigidity. The most common and dreaded morbidity is lymphedema. For clinically node-negative axilla, sentinel lymph node biopsy (SLNB) is now the procedure of choice, and patients with SLNB-negative axilla could be spared the morbidity of ALND. The incidence of postoperative lymphedema is considerably lower in sentinel lymph node biopsy cases. However, it has still not become negligible [3].

So, there is a need for mapping the axilla in terms of knowing the lymphatics that exclusively drain the breast and those draining the upper limb. Various techniques have evolved to map the first axillary lymph node that drains the breast (the sentinel lymph node). The technique involves giving the dye in the periareolar or peritumoral region and tracing it in the axilla. Dyes that are commonly used are methylene blue, isosulfan blue, fluorescein dye, indocyanine green (ICG), and radioactive colloid. The sensitivity of the dual-agent method (dye along with a radioactive colloid) is the highest. However, in expert hands, the dye-alone method is a feasible option when radioactive colloid is unavailable [3,4].

To map the lymphatics that drain the arm, axillary reverse mapping (ARM) was introduced in 2007 [5]. So, lymphatics draining the arm could be mapped and spared during axillary dissection. However, there are a few crossover lymphatics that drain both the breast and arm. These are held responsible for lymphedema caused even after axillary mapping and SLNB. There is limited data regarding crossover lymphatics between arm and breast lymphatics [6].

Against this background, a study is being contemplated to evaluate the role of the dual dye technique (using methylene blue and fluorescein dye) for SLNB and ARM to assess the axilla in patients with breast cancer [6].

## Materials and methods

The study of Chintamani et al. [3], observed that the sensitivity of SLNB was 86.6%. Using these values as a reference, the minimum required sample size with a desired precision of 20%, 80% power of study, and 5% level of significance is 29 patients. So the total sample size taken is 30.

The formula used is for testing the sensitivity and specificity of a single diagnostic test:

$N=\frac{[Z\frac{\alpha}{2}\sqrt{Se(1-Se)}+Z\beta\sqrt{Se_1(1-Se_1)}]^{2}}{(Se-Se_1)^{2}}$

where Se is the sensitivity; Z α/2 is the value of Z at a two-sided alpha error of 5%; Z β is the value of Z at a power of 80%.

Calculations

Sensitivity: H0: Se = 86.6 versus Se ≠ 86.6 (Se 1)

With a 95% confidence level and 80% power for detection of a difference of 20% from a Se of 86.6%, the sample size calculated is:

N = ((1.96*sqrt (.866*(1-.866)) + (.84*sqrt (.666*(1-.666))) 2 / (.2*.2) = 28.29 = 29 (approx)

Statistical analysis

Categorical variables will be presented in numbers and percentages (%), and continuous variables will be presented as mean ± SD and median. A diagnostic test will be used to calculate sensitivity, specificity, NPV, and PPV. A p-value of less than .05 will be considered significant. The data will be entered in an MS Excel spreadsheet, and analysis will be done using Statistical Package for Social Sciences (SPSS) version 21.0 (IBM Corp., Armonk, NY).

Type of study

This is a prospective observational cohort study.

Objectives

The objectives of this study are (1) to evaluate the identification rate, sensitivity, specificity, false negative rate, and accuracy of SLNB and the identification rate of arm lymphatics using dual dye (methylene blue and fluorescein) technique in breast cancer patients; (2) to identify the location of arm lymphatics in axilla with respect to intercostobrachial nerve; and (3) to study the incidence of seroma formation, flap necrosis, and postoperative lymphedema.

Inclusion criteria

All patients with histopathologically diagnosed breast cancer undergoing mastectomy or breast conservation surgery (BCS) with ALND are included.

Exclusion criteria

Pregnant ladies and patients who are sensitive to fluorescein or methylene blue were excluded from this study.

The study was conducted in the Department of General Surgery, Vardhman Mahavir Medical College & Safdarjung Hospital, New Delhi, India. Ethical clearance was obtained from the Institutional Ethics Committee before starting the study. All patients with histopathologically diagnosed breast cancer undergoing mastectomy or BCS with ALND were included in the study.

Sentinel lymph node biopsy

**Figure 1:**
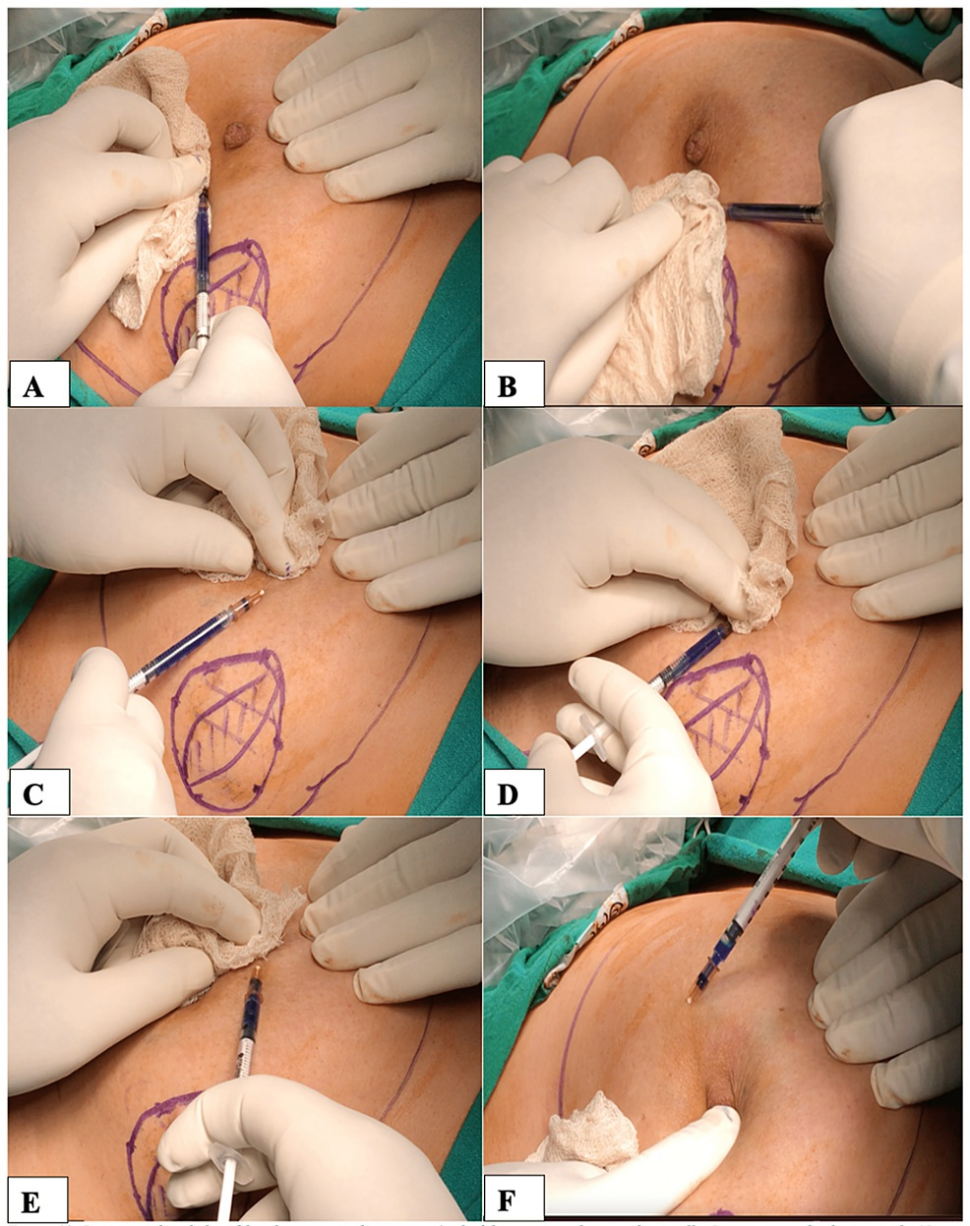
(A-F) Injection of methylene blue dye around the areola. 1 ml of dye is injected subcutaneously. Negative suction is always maintained while withdrawing the needle with the help of cotton gauze.

**Figure 2:**
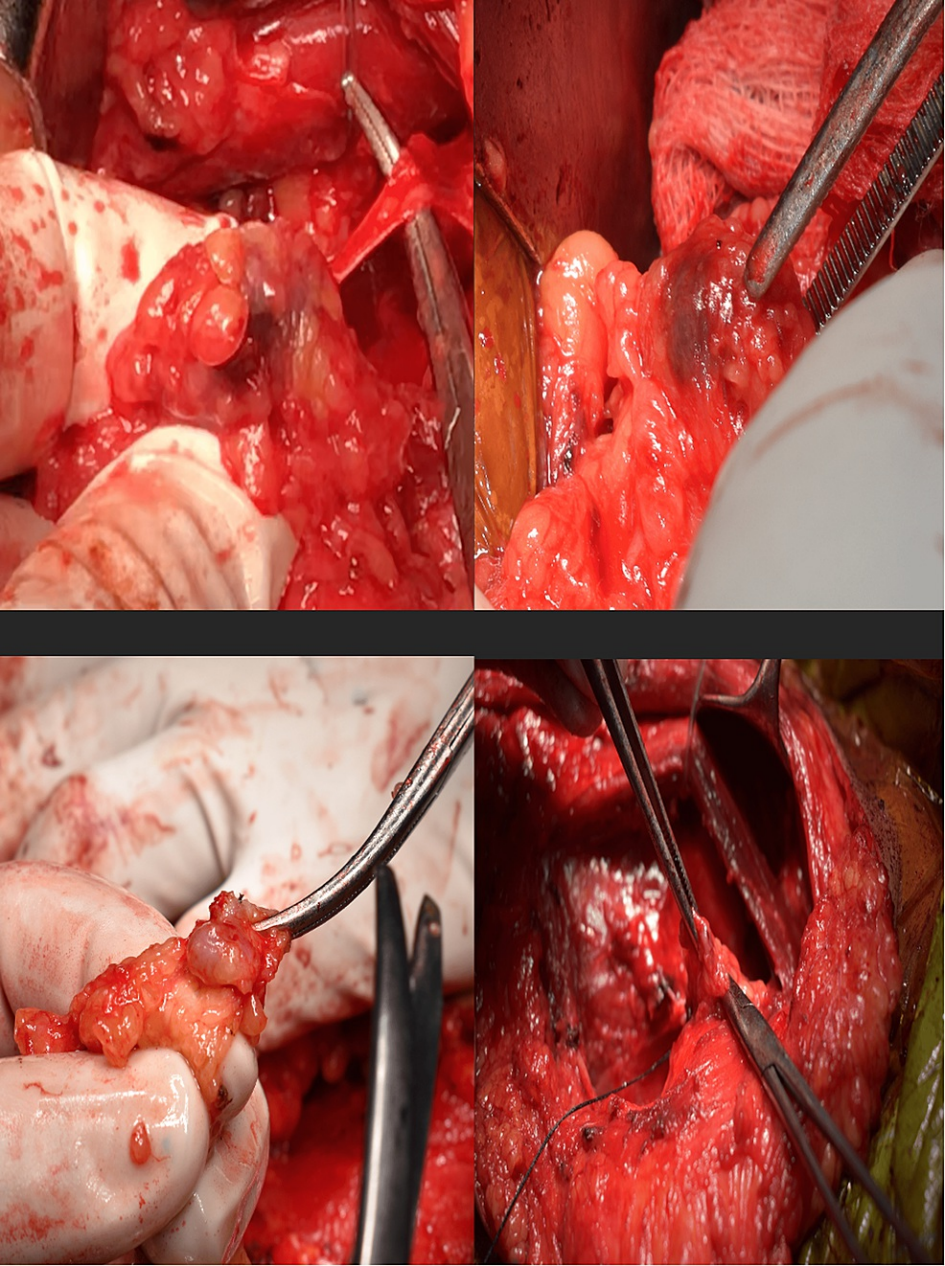
Sentinel lymph node (stained blue)

**Figure 3:**
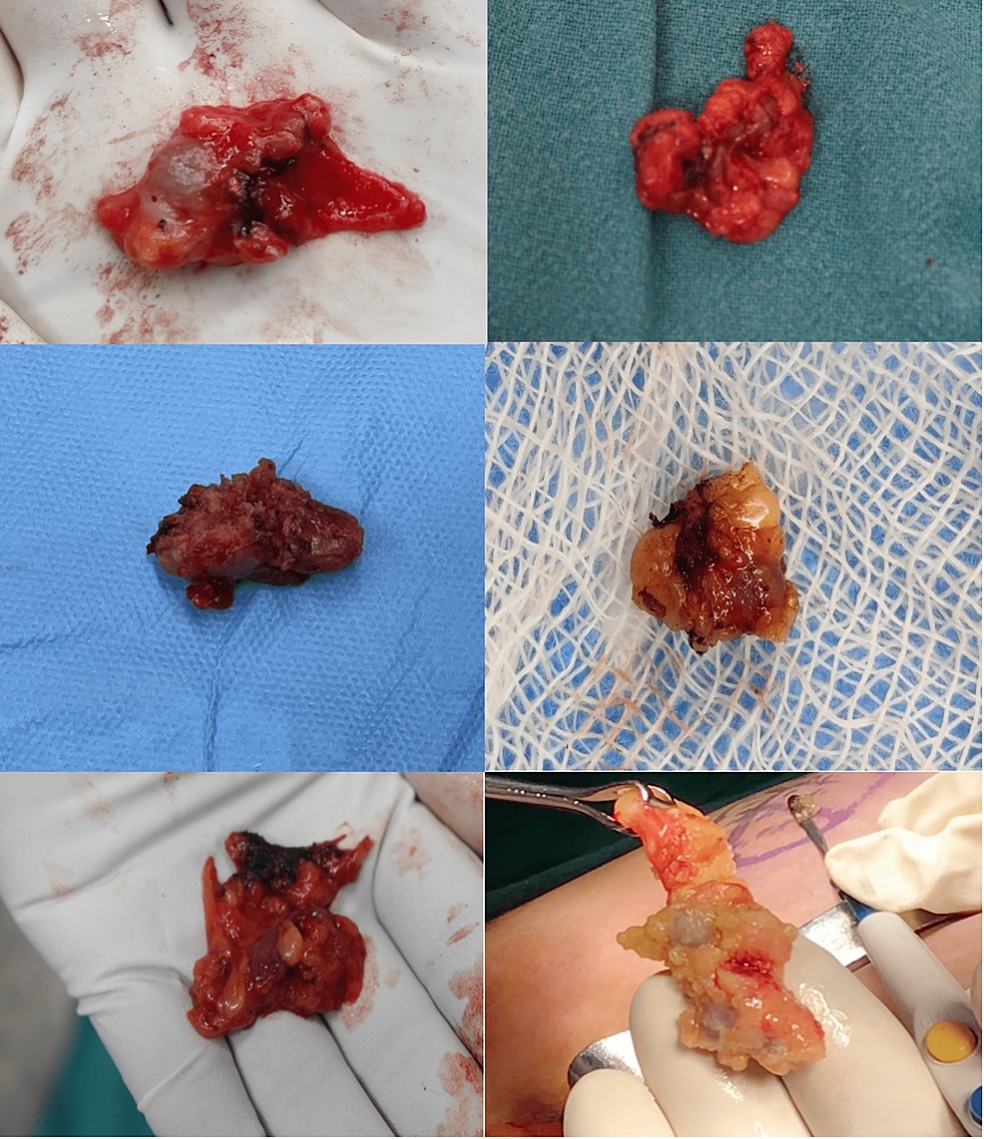
Nodes that appeared blue were dissected and sent for histopathological examination

**Figure 4:**
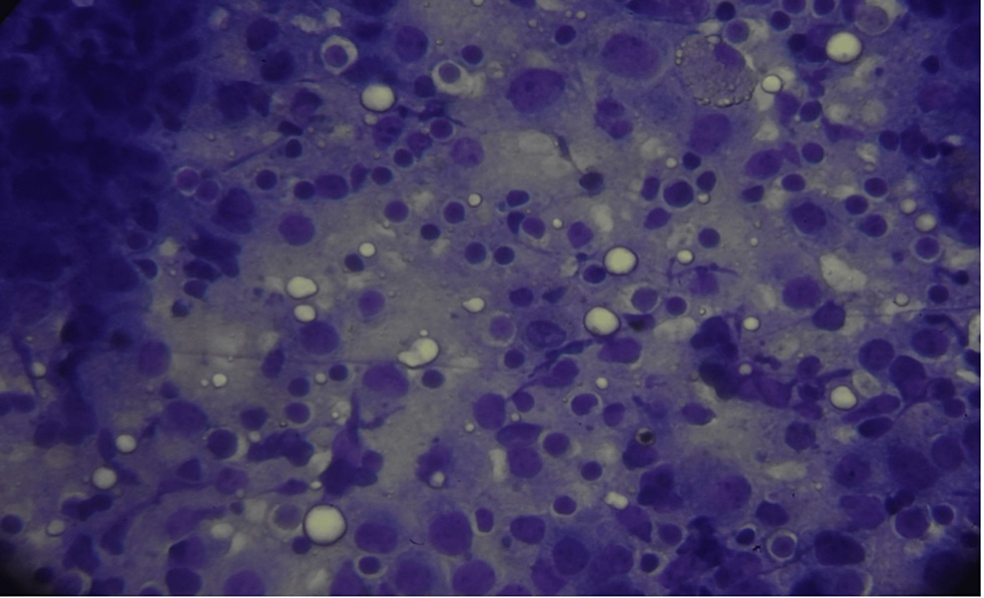
Imprint cytology of the sentinel lymph node

Intraoperatively, the tumor and incision were marked. A circumareolar injection of 1 ml of methylene blue dye was given. Dye was given subcutaneously in BCS (to prevent staining) and intradermally in modified radical mastectomy (MRM). Breast massage was done for 5 minutes. The upper and lower flaps were raised to the clavicle and inframammary crease, respectively, using an elliptical incision (Stewart incision). All lymph nodes that were stained blue were removed and intraoperatively subjected to pathological evaluation in the form of imprint cytology and frozen section (Figures 1-4).

Axillary reverse mapping

**Figure 5:**
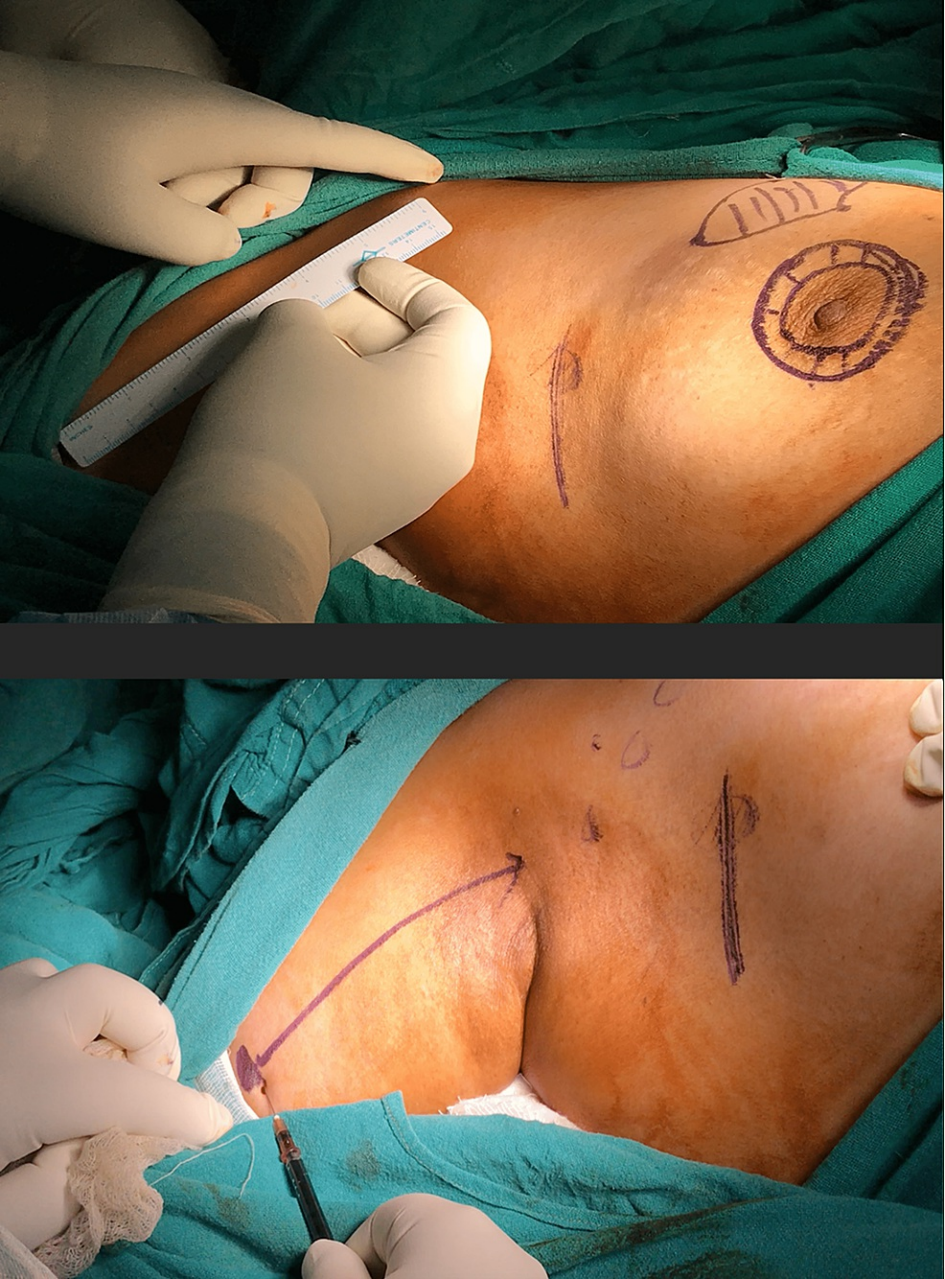
1 ml of fluorescein dye was injected subcutaneously

**Figure 6:**
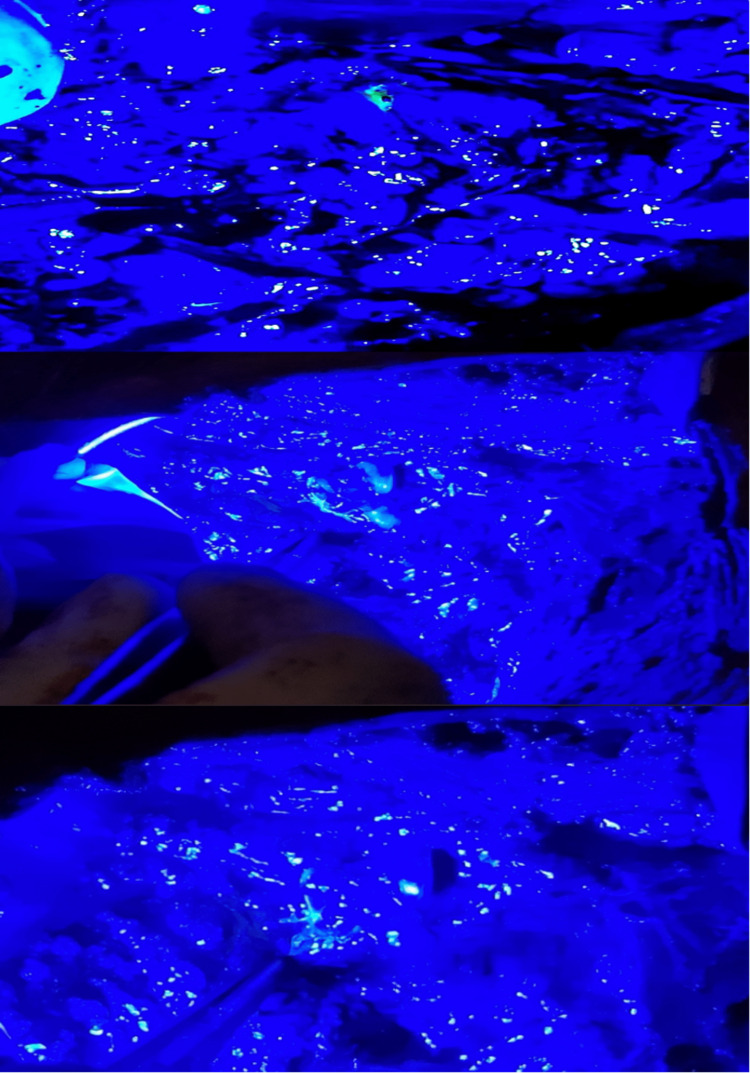
"Starry sky" appearance

**Figure 7:**
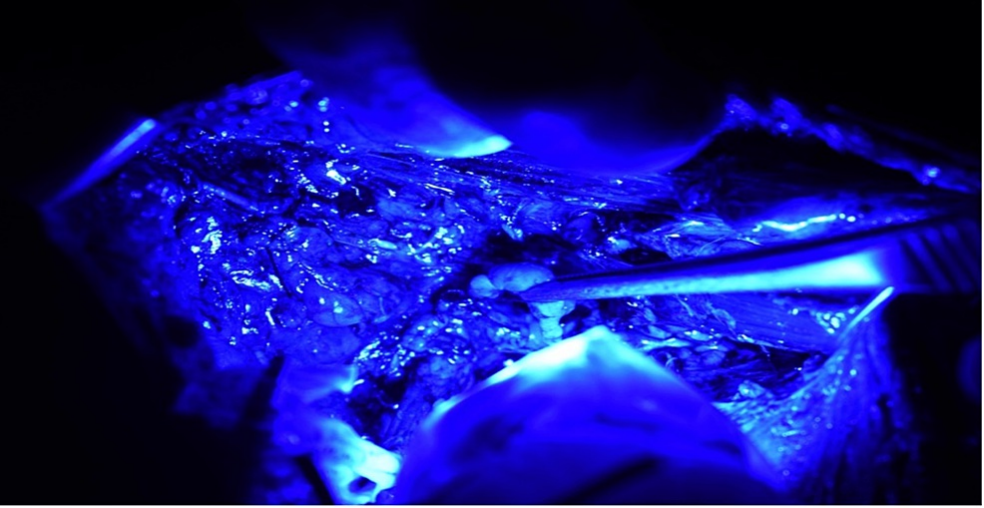
A fluorescent node held with plain forceps

**Figure 8:**
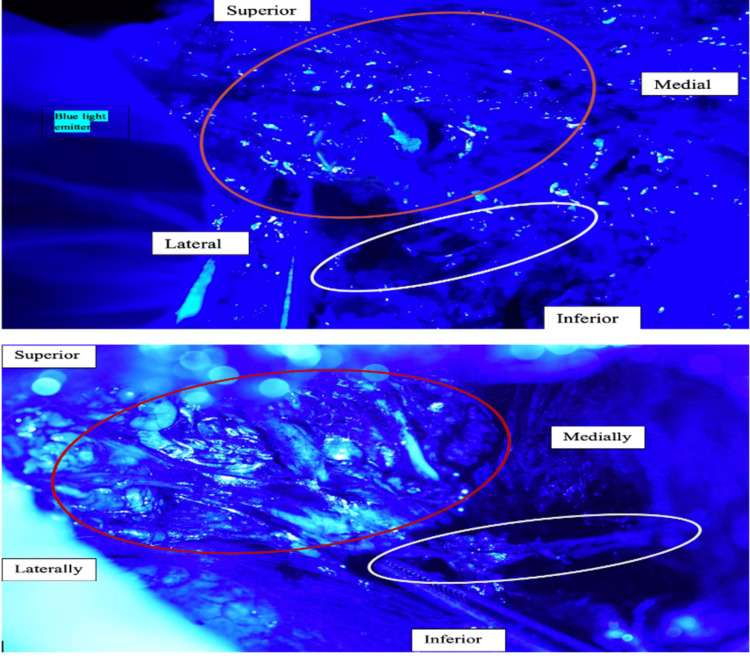
Relationship of ARM lymphatics (red circle) with the uppermost ICB nerve (white circle). The ARM lymphatics are predominantly visualized as superior to the ICB nerve. ARM: Axillary reverse mapping; ICB: Intercostobrachial.

**Figure 9:**
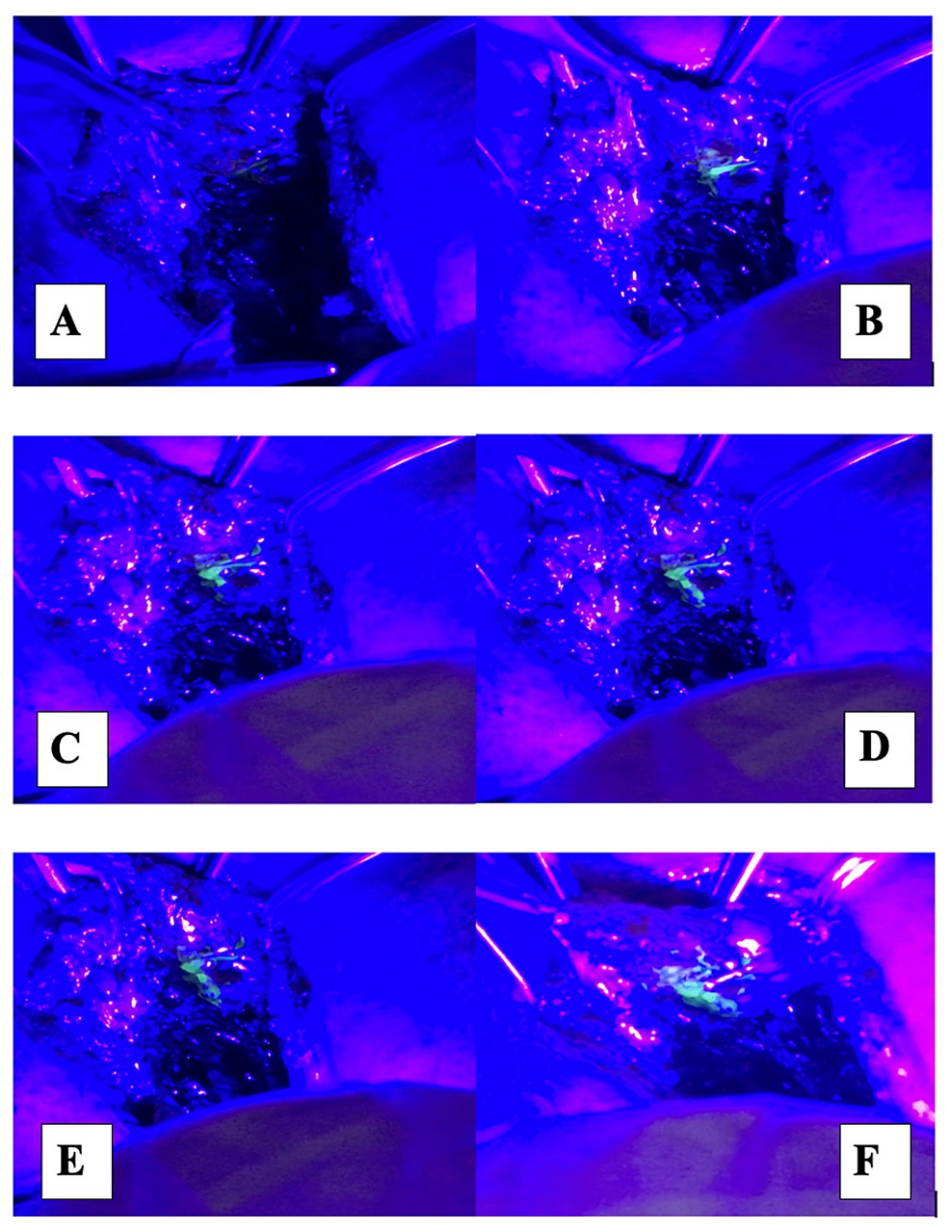
(A-F) Fluorescent spillage from ARM nodes into the surrounding lymphatics could be visualized by mobilizing it with a forceps ARM: Axillary reverse mapping.

**Figure 10:**
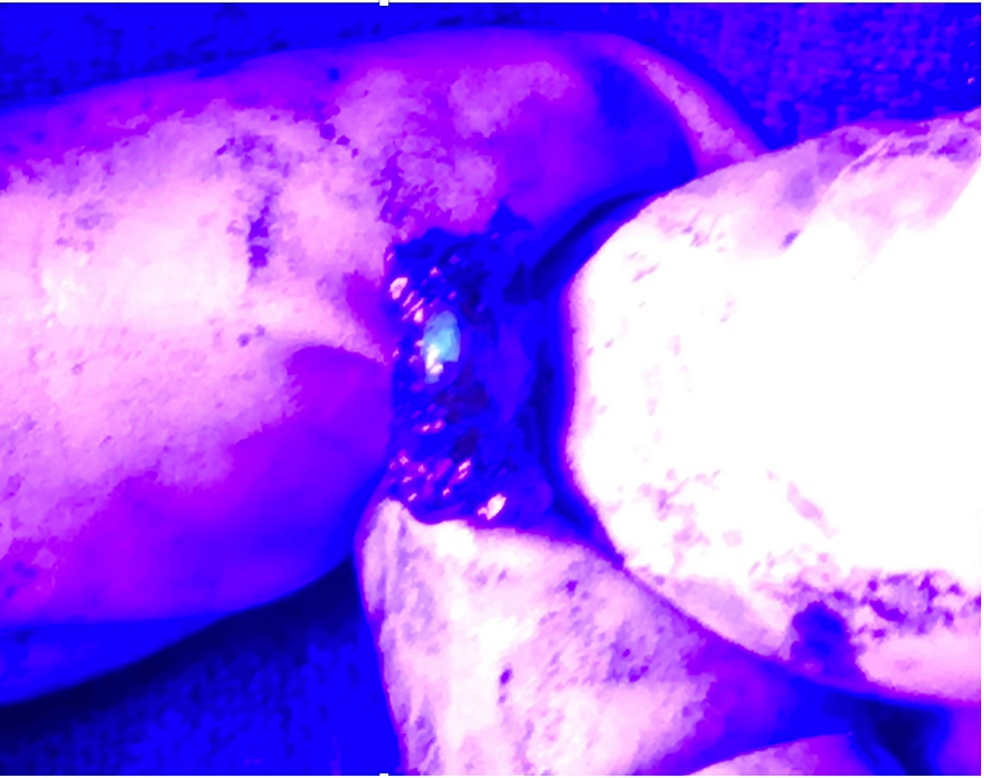
Arm node from axilla dissected and visualized under blue light. The node shows green fluorescence. The frozen section of the node did not have any metastatic deposits.

Intraoperatively, before making an incision, fluorescein dye (1 ml) was injected subcutaneously in the upper arm, 10 cm lateral to the acromion process. The injection site was massaged for five minutes. After SLNB and ALND, the axilla was visualized under blue light and recorded as digital pictures. Nodes and lymphatics that took up the dye produced greenish fluorescence under blue light. ALND was done in all cases, and clearance of axillary nodes was achieved in all cases (Figures 5-10).

**Figure 11:**
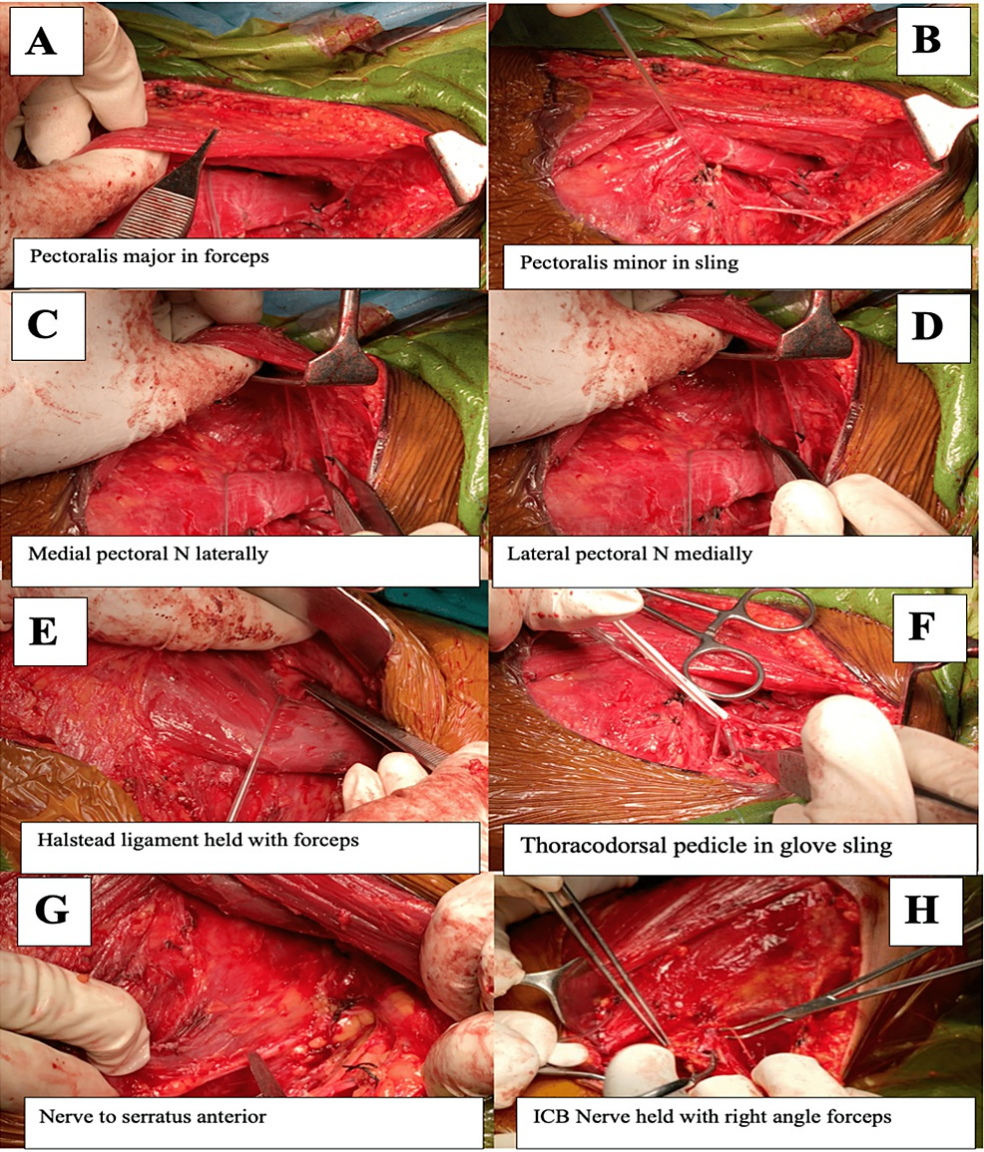
(A-H) Demonstrating structures after adequate mastectomy and axillary lymph node clearance ICB: Intercostobrachial.

Mastectomy was completed, and the specimen was sent for histopathological examination (HPE) after orientation. Upper and lower flaps were approximated after inserting the suction drain under half suction (Figure 11). Informed consent was taken from each patient regarding the ALND, ARM, SLNB, the extent of surgery, and digital photographic recording. Patients were provided with a detailed printed information sheet.

**Figure 12:**
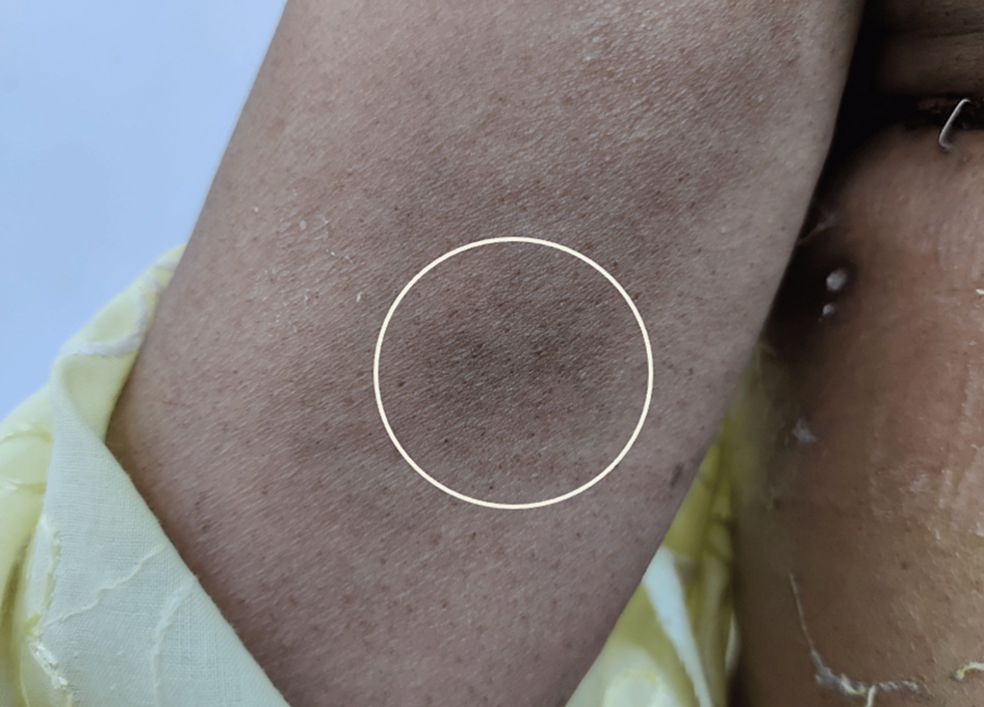
Foreign body granuloma at the site of fluorescein dye injection in the arm

In the postoperative period, all patients were regularly followed up and examined to look for complications in the form of seroma formation, flap necrosis, or foreign body granuloma at the site of dye injection (Figure 12).

## Results

The patients' age ranged from 28 to 71 years. The majority (36.7%) of the patients were 41-50 years old. The age was less than 30 years in only eight out of 30 patients and more than 50 years in 11 out of 30 patients. About 63.4% of the patients were below 50. The mean value of age (years) of study subjects was 48.20 SD 10.40, with a median (IQR) of 49.00 (40.00-56.75).

**Figure 13:**
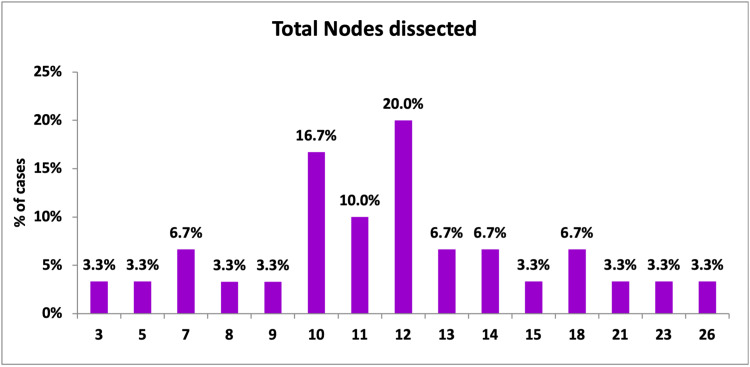
Total axillary nodes dissected per patient

All the patients included in the study were female. In the present study, the left side was predominantly involved among patients (56.70%). The study revealed that the total number of lymph nodes resected from patients ranged from 3 to 26. The variable with maximum frequency (i.e., mode) was 12 (20%). The mean ± SD was 12.13 ± 5.06. About 79.9% of patients attained optimum axillary dissection, i.e., a minimum of 10 nodes were dissected (Figure 13).

**Figure 14:**
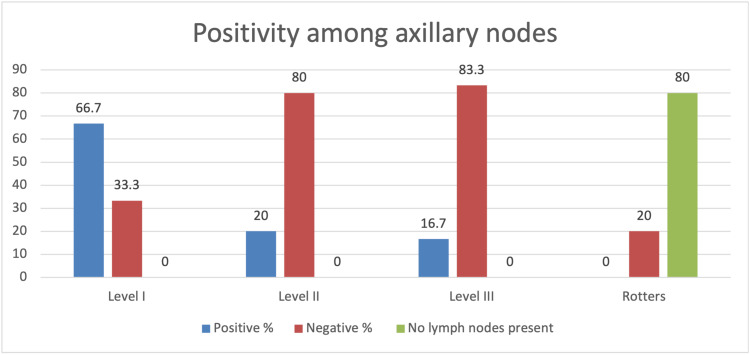
Positivity among axillary lymph nodes

Lymph node positivity rates among axillary nodes were level I (66.7%) > II (20%) > III (16.7%). Hundred percent of sentinel lymph nodes were identified in the level I group. Hence, a positive sentinel node would automatically become a positive level I group. Rotter nodes could not be identified in 80% of the specimens that were sent for histopathological examination. However, of those identified, none were found to be positive. At least one group (level I, II, or III) was found positive in 76.7% of total patients (Figure 14).

**Figure 15:**
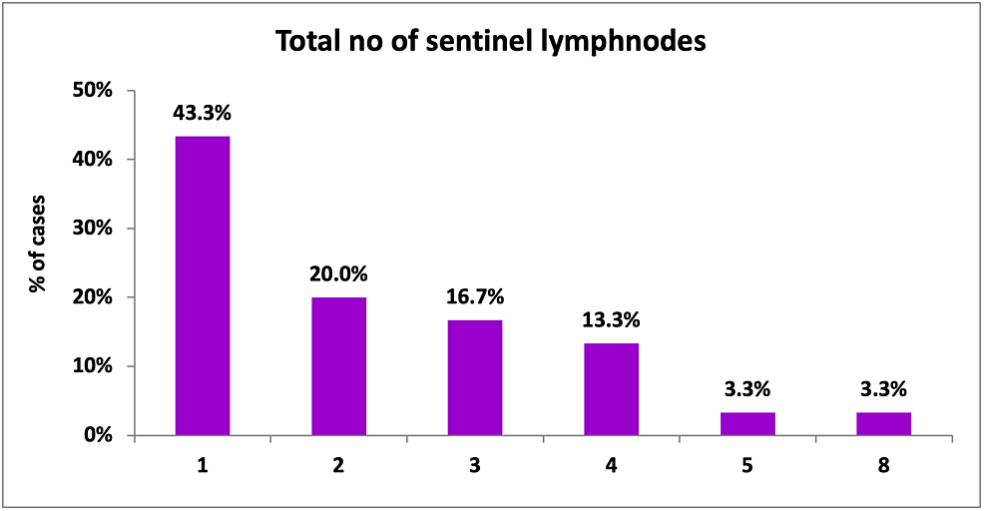
Total number of sentinel lymph nodes

Methylene blue-stained sentinel lymph nodes were identified in all patients (100%). The number of lymph nodes removed per patient ranged between 1 and 8. The mean ± SD was 2.30 ± 1.62. The majority of patients had one lymph node stained with blue dye (43.3%) (Figure 15).

**Figure 16:**
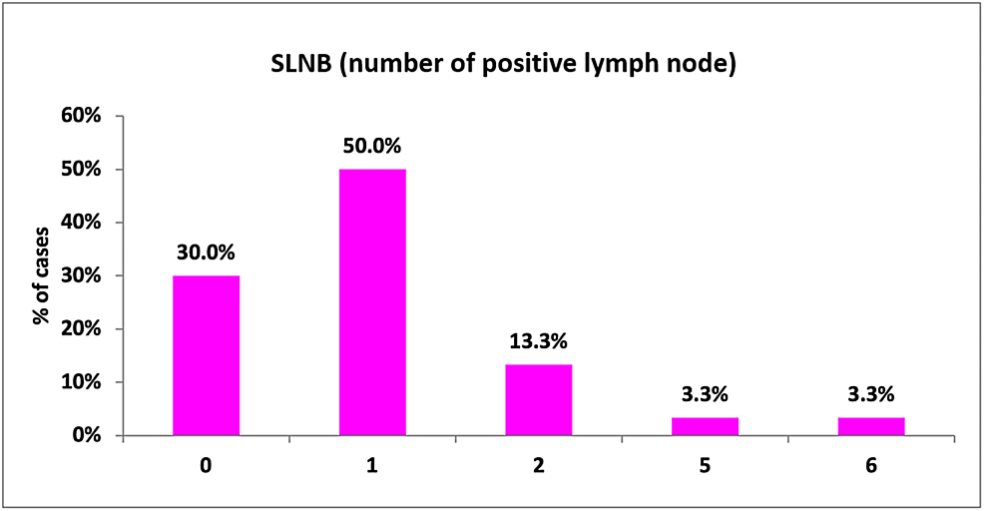
SLNB (number of positive lymph nodes) SLNB: Sentinel lymph node biopsy.

The sentinel lymph node was positive in 70% (21 out of 30) of the patients. About 50% (15 out of 30) of the patients had single-node positivity. However, the range of nodes with malignant change ranged from 1-6, with 3.3% of individuals having six positive sentinel lymph nodes (Figure 16).

**Figure 17:**
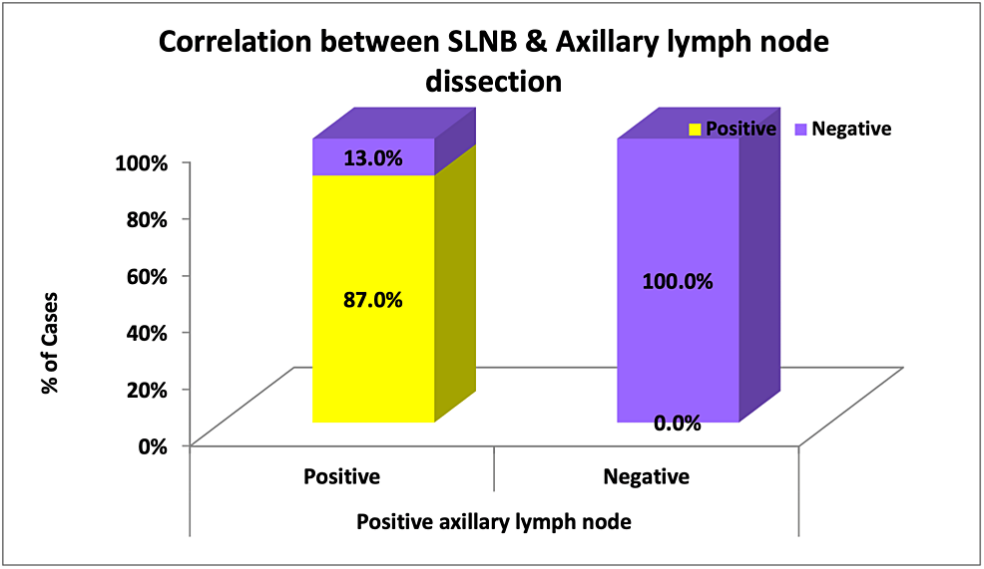
Correlation between SLNB and axillary lymph node dissection SLNB: Sentinel lymph node.

From the 2x2 table (Table 1), SLNB can be compared with the gold standard of staging axilla, i.e., axillary lymph node dissection (Figure 17).

**Table 1:** Correlation between SLNB and axillary lymph node dissection SLNB: Sentinel lymph node biopsy.

SLNB	Positive axillary lymph node (SLNB considered as level 1 LN)				p-value
	Positive		Negative		
	Frequency	%	Frequency	%	
Positive	20 (TP)	87.0%	0 (FP)	0.0%	<0.001
Negative	3 (FN)	13.0%	7 (TN)	100.0%	
Total	23	100%	7	100%	

From this, the following values could be calculated:

Sensitivity of SLN = True Positives/(True Positives + False Negatives) = 87%

False Negative Rate = False Negatives/(False Negatives + True Positives) = 13%

Negative Predictive Value = True Negative/(True Negative + False Negative) = 70%

Specificity of SLN = True Negatives/(True Negatives + False Positives) = 100%

False Positive Rate = False Positives/(False Positives +True Negatives) = 0%

Positive Predictive Value = True Positive/(True Positive + False Positive) = 100%

Accuracy = True Positive + True Negative/No. of Patients With Successfully Identified SLN = 90%

**Figure 18:**
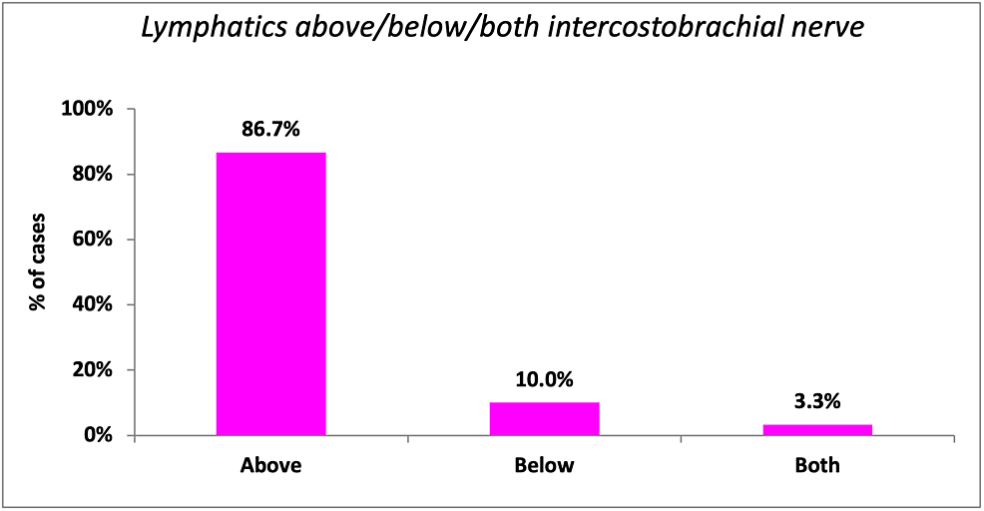
Association of ARM lymphatics with respect to intercostobrachial nerve ARM: Axillary reverse mapping.

ARM lymphatics were identified in the axilla in all 30 patients. ARM lymphatics were located exclusively above the uppermost intercostal nerve in 86.7% of patients as shown in Figure 18.

Patients were followed up in the postoperative period of at least one year, for complications associated with mastectomy and axillary dissection like flap necrosis, seroma, and lymphedema. Two out of 30 patients (6.7%) developed necrosis of the flap and required intervention in the form of debridement. One patient (3.3%) developed granuloma at the site of injection of fluorescein dye, which was relieved on its own two months postoperatively (Figure 12).

## Discussion

Surgical management used to be radical, which resulted in a high incidence of postoperative complications. The current study uses a dual dye technique that uses methylene blue dye for identifying sentinel nodes and fluorescent dye to trace lymphatics draining the arm.

Sentinel lymph node biopsy

SLNB is ideally done with dual agents, including blue dye and a radiotracer. However, recent studies show that single-dye usage has a similar identification rate and efficacy [6,7]. We have limited studies of sentinel biopsy done along with ARM, which validates the purpose of this study.

Distribution and Number of Sentinel Lymph Nodes and Axillary Lymph Nodes

In the present study, all patients had sentinel lymph nodes at level I. The average number of axillary and sentinel lymph nodes dissected was 12.13 and 2.3, respectively. The average number of axillary nodes dissected was more than 10, i.e., more than the minimum number required for optimum axillary dissection.

In the study by Nandu et al. [7], 35 patients underwent a SLNB. Methylene blue dye was injected at 10 mg/ml intralesionally and perilesionally for 20 minutes prior to surgery. All patients underwent MRM. Blue nodes were visible in level I in 88.6% of patients, with the rest divided among level II and the internal mammary group. The mean number of axillary nodes and sentinel nodes dissected was 17.34 and 1.8, respectively.

In the study by Somashekhar et al. [8], 100 patients underwent SLNB. Methylene blue dye and radioactive Tc 99m sulfur colloid were used. About 0.2 ml of radioactive technetium 99m (Tc99m) sulfur colloid was injected peritumorally in all four quadrants and intradermally in the skin anterior to the lump (total of 1 ml) two hours prior to surgery; 1 ml of methylene blue dye (10%) was injected peritumorally in all four quadrants (total of 4 ml) 20 minutes prior to surgery. Blue-stained nodes and/or nodes with a high count and audible tone (the hottest node) as identified by a hand-held gamma probe were excised and sent for HPE. Blue nodes were visible in level I in 86% of patients, with the rest divided among level II and the internal mammary group. The mean number of axillary nodes and sentinel nodes dissected was 16 and 2 (Table 2).

**Table 2:** Distribution and number of sentinel lymph nodes and axillary lymph nodes

	Present study	Nandu et al. [7]	Somashekhar et al. [8]
Sentinel lymph node, level (%)			
I	100	88.6	86
II	0	5.7	6
I and II	0	2.9	6
Internal mammary node	0	2.86	2
Mean number of axillary lymph nodes dissected	12.13 ± 5.06 (3–26)	17.34	16
Mean number of sentinel lymph nodes dissected	2.30 ± 1.62 (1–8)	1.8	2

Comparison of SLNB Among Studies Using a Single Agent 

In the present study, identification rates and specificity were estimated at 100%, which is higher than any available study on SLNB using a single agent. Sensitivity (87%), false negative rate (13%), and accuracy (90%) were comparable to other studies.

In the study by Chintamani et al. [3], a single-dye study was done with methylene blue dye in the same institution. This study was done on patients who received neoadjuvant chemotherapy (NACT). All diagnostic parameters given in Table 3 were comparable to the present study. However, the number of patients with positive axillary burden (13/30) was low as compared to the present study. This was probably due to decreased axillary burden following NACT in these patients.

**Table 3:** Comparison of SLNB among studies using a single agent SLNB: Sentinel lymph node biopsy.

Sentinel lymph node	Present study	Chintamani et al. [3]	Mamounas et al. [9]	McMasters et al. [10]	Gupta et al. [11]	Nandu et al. [7]
Identification rate (%)	100	100	78	86	100	100
Sensitivity (%)	87	86.67			75	90.5
Specificity (%)	100				95.65	85.7
False negative rate (%)	13	13.33	14	12	8.6	
Accuracy (%)	90	93			90	88.57

Mamounas et al. [9] and McMasters et al. [10] have done SLNB with the help of methylene blue dye alone. These studies had low identification rates (78% and 86%, respectively) but comparable false negative rates (14% and 12%).

Gupta et al. [11] studied 60 patients with early breast cancer. His patients were divided into two groups, A and B, with 30 patients each. One group underwent SLNB with methylene blue alone, whereas the other group underwent SLNB with methylene blue and a radioactive colloid. Diagnostic statistics for both groups are given in Tables 3 and 4. The study had a better false negative rate (8.6%), but the rest of the parameters are comparable to the present study. In the study by Nandu et al. [7], 35 patients with breast cancer with a node-negative axilla underwent SLNB. Diagnostic statistics were similar to the present study, with higher sensitivity (90.5%) and lower specificity (85.7%).

**Table 4:** Comparison of diagnostic statistics of SLNB in dual-agent studies SLNB: Sentinel lymph node biopsy.

Sentinel lymph node	Present study	Gupta et al. [11]	Somashekhar et al. [8]	Tafra et al. [12]	Mamounas et al. [9]	McMasters et al. [10]
Identification rate (%)	100	100	100	87	88	90
Sensitivity (%)	87	83.33	96.2			
Specificity (%)	100	91.67	100			
False negative rate (%)	13	4.3	3.7	13	9	6
False positive rate (%)	0	28.7	0			
Accuracy (%)	90	90				

Comparison of Diagnostic Statistics of SLNB in Dual-Agent Studies 

Gupta et al. [11], Somashekhar et al. [8], Tafra et al. [12], Mamounas et al. [9], and McMasters et al. [10] used blue dye along with radiotracers to identify sentinel lymph nodes. The studies by Gupta et al. [11], Mamounas et al. [9], and McMasters et al. [10] also included sentinel lymph node identification with the help of a single agent (Table 4).

The present study, with the use of a single agent, has results comparable to those of dual-agent studies for tracing sentinel lymph nodes. The present study has the best identification rate of 100%, along with Gupta et al. [11] and Somashekhar et al. [8], the best specificity of 100%, and the lowest false positivity rate of 0%, along with Somashekhar et al. [8]. The false negative rate (3.7%) was the lowest, and sensitivity (96.2) was the highest in the study by Somashekhar et al. [8].

Axillary reverse mapping

Comparison of Identification Rates 

The present study has an identification rate of 100% for ARM with fluorescein dye. ARM was done after ALND in all 30 patients. In the meta-analysis by Han et al. [6], 24 prospective studies were included. Eleven studies reported ARM along with SLNB, and 18 studies reported ARM during ALND. The identification rate of arm lymphatics was done with the help of blue dye, a combination of blue dye and radiotracer, and fluorescein dye alone. The identification rate was highest with fluorescein dye (92.7%). Studies with ARM done along with SLNB revealed an overall identification rate of 38.2%, which is far lower as compared to ARM done along with ALND. The crossover rate among SLN and ARM nodes was 19.6%. ARM nodes were involved in 16.9% of cases. The pooled incidence of lymphedema was 4.1% among patients undergoing the ARM procedure (Table 5).

**Table 5:** ARM: Comparison of identification rates ARM: Axillary reverse mapping; ALND: Axillary lymph node dissection; SLNB: Sentinel lymph node biopsy.

	Present study (fluorescein dye) (ALND)	Han et al. [6]				
		ALND				SLNB
		Blue dye	Blue dye + Radio-isotope	Fluorescence	Overall	Overall
Identification rate (%)	100	78.4	88.5	92.7	82.8	38.2

Comparison of Identification Rates Among Single-Agent Studies 

In the study by Noguchi et al. [13], 131 patients were included. Clinically, node-negative patients (34/131) underwent SLNB, while the rest of the patients (97/131) who had enlarged axillary lymph nodes underwent ALND. SLNB was done using both methylene blue dye and radiotracers. Patients with positive SLNB subsequently underwent ALND and ARM. ARM nodes were removed separately in all the patients. The identification rate of ARM was 85% among patients who underwent ALND and 43% among patients who underwent SLNB.

In the study by Ikeda et al. [14], ARM lymphatics were traced during ICG with an infrared camera. The identification rate in the present study was 82%. The present study, which was based on fluorescein dye and blue light, has a 100% identification rate for arm lymphatics using ARM. This is better than using ICG and infrared cameras, which are also expensive and not easily available (Table 6).

**Table 6:** ARM: Comparison of identification rates among single-agent studies (fluorescein dye + blue light versus indocyanine green + infrared camera) ARM: Axillary reverse mapping.

	Present study	Noguchi et al. [13]	Ikeda et al. [14]
Identification rate (%)	100	85%	82

Comparison of Location With Respect to the Uppermost ICBN 

In the study by Ikeda et al. [15], the axilla was divided into four zones (A, B, C, and D) based on the intercostobrachial nerve and thoracodorsal pedicle. Above the ICB nerve were zones A and B, whereas C and D were below the ICB nerve. ARM was done with the help of an ICG and an infrared camera. Arm lymphatics were predominantly above the ICB nerve (88%) in the axilla. The results of the present study are comparable (above ICBN: 86.7%). In a study by Li et al. [16], the rate of lymphedema among patients who underwent partial ALND, inferior to the uppermost intercostobrachial nerve, was considerably less (Table 7).

**Table 7:** ARM: Comparison of location with respect to the uppermost ICBN ARM: Axillary reverse mapping; ICBN: Intercostobrachial nerve.

Location of ARM nodes with respect to intercostobrachial nerve (%)	Present study	Ikeda et al. [15]
Above	86.7	88
Below	10	12
Both	3.3	0

Limitations of the study

Despite the aforementioned advantages, the study is subject to a few constraints. The majority of these findings can be attributed to the limited size of the sample and the absence of a control group. The average number of lymph nodes dissected in this study was found to be optimal, but it was lower compared to the findings reported in other studies [7,8].

The limited sample size hindered the ability to compare diagnostic data of SLNB and ALND across several groups, including early versus locally advanced breast cancer, those who underwent upfront surgery versus post-neoadjuvant chemotherapy, and those who underwent SLNB alone versus ALND alone.

Even though the specificity of the SLNB was the same in this study, the rate of false negatives was higher than in studies that used a dual-agent approach [8,11]. The oncological safety of ARM was not validated due to the absence of lymphatic sampling in all patients. The duration of the postoperative follow-up period may be extended in order to identify delayed complications, such as recurrence.

## Conclusions

In our study population, 63.4% of the patients were below 50 years of age, indicating a changing trend of demographic shift toward younger age groups. Mapping sentinel lymph nodes with a single agent like methylene blue dye has results comparable to studies that used dual agents (dye along with a radiotracer). The present study has produced comparable or better outcomes as compared to previous studies, which were done with single agents.

Arm lymphatics in the axilla could be identified in 100% of the patients, which was comparable to or better than previously conducted studies. Arm lymphatics were predominantly located superior to the uppermost intercostobrachial nerve. This observation aligns with prior research that has reported a low incidence of lymphedema among individuals who have undergone partial ALND, namely below the uppermost intercostobrachial nerve. The use of fluorescein sodium and blue light in the research is advantageous due to their affordability and widespread accessibility, in contrast to alternative fluorescent agents such as ICG with gamma cameras. The utilization of the uppermost intercostobrachial nerve as the superior boundary for ALND can be considered a potentially advantageous surgical refinement aimed at decreasing the occurrence of lymphedema in the upper extremities in a selected group of patients.
